# Effects of Simulated 5-Ion Galactic Cosmic Radiation on Function and Structure of the Mouse Heart

**DOI:** 10.3390/life13030795

**Published:** 2023-03-15

**Authors:** Ashley S. Nemec-Bakk, Vijayalakshmi Sridharan, Parth Desai, Reid D. Landes, Barry Hart, Antiño R. Allen, Marjan Boerma

**Affiliations:** 1 Division of Radiation Health, University of Arkansas for Medical Sciences, Little Rock, AR 72205, USA; 2 Department of Biostatistics, University of Arkansas for Medical Sciences, Little Rock, AR 72205, USA; 3 Innovation Pathways, LLC of Palo Alto, Palo Alto, CA 94301, USA

**Keywords:** galactic cosmic radiation, mouse model, cardiovascular system, degenerative tissue effects, transforming growth factor-β

## Abstract

Missions into deep space will expose astronauts to the harsh space environment, and the degenerative tissue effects of space radiation are largely unknown. To assess the risks, in this study, male BALB/c mice were exposed to 500 mGy 5-ion simulated GCR (GCRsim) at the NASA Space Radiation Laboratory. In addition, male and female CD1 mice were exposed to GCRsim and administered a diet containing Transforming Growth Factor-beta (TGF-β)RI kinase (ALK5) inhibitor IPW-5371 as a potential countermeasure. An ultrasound was performed to investigate cardiac function. Cardiac tissue was collected to determine collagen deposition, the density of the capillary network, and the expression of the immune mediator toll-like receptor 4 (TLR4) and immune cell markers CD2, CD4, and CD45. In male BALB/c mice, the only significant effects of GCRsim were an increase in the CD2 and TLR4 markers. In male CD1 mice, GCRsim caused a significant increase in total collagens and a decrease in the expression of TLR4, both of which were mitigated by the TGF-β inhibitor diet. In female CD1 mice, GCRsim caused an increase in the number of capillaries per tissue area in the ventricles, which may be explained by the decrease in the left ventricular mass. However, this increase was not mitigated by TGF-β inhibition. In both male and female CD1 mice, the combination of GCRsim and TGF-β inhibition caused changes in left ventricular immune cell markers that were not seen with GCRsim alone. These data suggest that GCRsim results in minor changes to cardiac tissue in both an inbred and outbred mouse strain. While there were few GCRsim effects to be mitigated, results from the combination of GCRsim and the TGF-β inhibitor do point to a role for TGF-β in maintaining markers of immune cells in the heart after exposure to GCR.

## 1. Introduction

Future missions into deep space will leave astronauts exposed to space radiation at levels that would not be experienced in low Earth orbit (LEO). Since few astronauts have left LEO thus far, the health risks of long-distance space travel are estimated from ground-based models or from missions on the International Space Station. Previous ground-based studies investigating the effects of simulated space radiation on the cardiovascular system have utilized high atomic weight, high energy (HZE) radiation in the form of single or fractionated exposures to one ion [1,2,3,4,5,6]. These previous rodent studies have shown alterations in cardiac function [7], cardiac inflammation [4], and vascular stiffness and endothelial dysfunction [8]. Recent studies have also demonstrated that HZE exposure resulted in cellular changes that could lead to cardiovascular disease [9,10]. Our previous studies utilizing single ion beams from NASA’s Space Radiation Laboratory (NSRL) have demonstrated minor changes in cardiac form and function [1,2], and the cardiac changes may be different between sexes [6]. Since the radiation environment in deep space consists of multiple ions at varying fluences, it is important to determine the health risks associated with multi-ion exposures. New advancements at NSRL have enabled the production of fields of mixed ions to better represent the deep space radiation environment [11]. This study used NSRL’s 5-ion simulated galactic cosmic ray (GCRsim) beam [12] to evaluate the cardiac structure and function in murine models. 

Mouse models allow us to determine the risks of high linear energy transfer (LET) radiation, but there is debate on which models best represent the human health effects of space radiation, and data from mice do not always translate to humans [13]. One approach to increase the translatability of mouse model data is to include both inbred and outbred mouse strains to include more diversity in the genetic background [14,15].

Cardiovascular diseases associated with radiation exposure are often late effects, and the threshold dose for the adverse cardiovascular effects is still uncertain [16,17]. Since few astronauts have traveled beyond LEO, the cardiovascular effects of GCR exposure are not well understood. However, as mentioned above, studies in small animal models of single high energy ion exposures such as ^56^Fe, ^28^Si, ^16^O and others have shown mild cardiac and vascular remodeling and dysfunction several weeks to months after exposure [2,4,7,9]. In addition, there is concern about the negative effects of microgravity on the cardiovascular system due to fluid shifts [18,19]. Therefore, finding countermeasures that may reduce cardiovascular disease risk in astronauts is one of NASA’s top priorities. There are only a few countermeasures against the effects of low-LET radiation exposures commercially available, and whether they are effective against cardiovascular effects from space radiation are not always clear [20,21]. Therefore, research is needed to identify potential countermeasures against space radiation. Transforming growth factor-β (TGF-β) is involved in many cellular pathways and regulates cellular processes including cell proliferation, fibrosis, and inflammatory regulation [22,23,24,25]. Dysregulation of this growth factor or its signaling pathways contribute to adverse remodeling in cardiovascular disease including hypertension [26], atherosclerosis [24], and heart failure [27]. Previous studies have also implicated TGF-β in adverse remodeling in the heart after high-dose low-LET radiation exposure [28,29,30]. Therefore, the inhibition of the TGF-β signaling pathway could be an interesting target for a countermeasure against the biological effects of HZE radiation.

This study was designed to test the effects of NSRL’s 5-ion GCRsim exposure on the heart of an inbred and outbred mouse strain and investigate whether TGF-β inhibition is an effective countermeasure against cardiovascular effects of GCR. Mice were irradiated at the age of 6 or 9 months to simulate exposure at middle age.

## 2. Materials and Methods

Male BALB/c mice were purchased from The Jackson Laboratory (Bar Harbor, ME, USA) at p21 and housed 5 per cage at the University of Arkansas for Medical Sciences (UAMS) until they were 6 months old. Mice were fed the Picolab Select Rodent Diet 50 IF/6F 5V5R. They had access to chow and water ad libitum and were housed on a 12:12 h light:dark cycle.

Male and female CD1 mice were also obtained from The Jackson Laboratory at p21 and housed 5 per cage at UAMS until they were 9 months old. CD1 mice also had constant access to water and chow while housed on a 12:12 h light:dark cycle.

At 6 or 9 months of age, mice were transported to Brookhaven National Laboratory (BNL; Upton, NY, USA) by overnight air transportation (World Courier, London, UK), where they were housed on a 12:12 h light:dark cycle. After a 1-week acclimation period at BNL, mice were exposed to whole-body simplified simulated 5-ion GCR. Unanesthetized mice were placed in individualized clear and well-ventilated holders and positioned in the NSRL beam line. The five ions were given in six consecutive beams as follows: protons (1 GeV, 175 mGy), ^28^Si (600 MeV/n, 5 mGy), ^4^He (250 MeV/n, 90 mGy), ^16^O (350 MeV/n, 30 mGy), ^56^Fe (600 MeV/n, 5 mGy), and protons (250 MeV, 195 mGy) to a total dose of 500 mGy over about 4.5 h. Sham-irradiated mice were treated the same as the irradiated groups but were not exposed to radiation. Radiation dosimetry was performed by the NSRL physics team.

Within the first week after irradiation or sham treatment, mice were returned to UAMS by overnight air transportation and housed for 15 weeks, 20 weeks (BALB/c mice), or 12 weeks (CD1 mice). 

Male and female CD1 mice were placed on a diet containing 30 mg/kg/day of TGF-β receptor 1 antagonist inhibitor, IPW-5371 (Innovation Pathways, Palo Alto, CA, USA) [31] 48 h before GCRsim exposure until 12 weeks after GCRsim. There were four groups of mice in this study: Control diet, 0 mGy; Control diet, 500 mGy; TGF-β inhibitor diet, 0 mGy; TGF-β inhibitor diet, 500 mGy.

At 12 weeks (CD1 mice) or 15 weeks (BALB/c mice) after GCRsim, a high-resolution ultrasound was performed to investigate cardiac function. Anesthesia is known to alter cardiac physiology [32], therefore the mice were under anesthesia for the shortest possible time (~10 min) during the procedure. Ultrasounds were performed as previously described [1]. All data collected using ultrasonography are described in the Supplementary Materials, Table S1.

At 12 weeks (CD1 mice) and 15 and 20 weeks (BALB/c mice) after GCRsim, cardiac tissue was collected. Mice were anesthetized with 3% isoflurane. Cardiac tissue was collected, rinsed in PBS, and cut into 2 longitudinal halves. One half of the heart was fixed in methanol Carnoy’s solution (60% methanol, 30% chloroform, 10% acetic acid) for 24 h and embedded in paraffin for further analysis. The left ventricle of the other half of the heart was divided into four samples before snap-freezing the tissues in liquid nitrogen.

Total RNA was isolated using Ultraspec^TM^ RNA reagent (Biotecx Laboratories, Houston, TX, USA) using a motorized homogenizer. The samples were treated with RQ-DNAse I (Promega, Madison, WI, USA) and incubated at 37 °C for 30 min. cDNA was synthesized using the High-Capacity cDNA Archive Kit^TM^ (Applied Biosystems, Foster City, CA, USA). Steady-state mRNA levels were measured with real-time quantitative PCR (TaqMan^TM^) using the ABI Prism 7500 Fast system, TaqMan Universal PCR Mastermix, and the following pre-designed TaqMan Gene Expression Assay^TM^: TGF-β1 (Mm01178820_m1). Expression of TGF-β was normalized to the internal control gene 18S (Hs99999901_s1) and expressed as fold change according to the ΔΔCT method (All Applied Biosystems).

Collagen deposition was determined by using 5-µm longitudinal sections of the hearts, which were rehydrated and incubated in Sirius Red supplemented with Fast Green. Stained hearts were visualized using a ScanScope CS2 slide scanner and analyzed with ImageScope 12 software (Leica Biosystems, Wetzlar, Germany). The relative tissue area of collagens was calculated as the red-stained area expressed as a percentage of the total tissue area of each section.

Mast cell density was determined by incubated cardiac sections in 0.5% Toluidine Blue in 0.5 N HCl for 3 days at room temperature, followed by 0.7 N HCl for 10 min. Eosin was used as a counterstain. Cardiac sections were evaluated using an Axioskop transmitted-light microscope (Carl Zeiss, Oberkochen, Germany). Mast cell numbers were determined by a researcher blinded to animal treatment, and mast cell numbers were divided by the total tissue area of each section as determined with ImageScope 12 software (Leica Biosystems).

To quantify the density of the capillary network, biotinylated tomato lectin was used to identify the microvessels and stained with the Vectastain ABC-HRP kit (Vector Laboratories, Newark, CA, USA). Using an Axioskop transmitted-light microscope (Carl Zeiss) at 40× magnification, five image areas of the myocardium in the ventricles were selected. Capillaries were counted and divided by the total tissue area.

An immunoblot analysis was performed on frozen left ventricle tissue samples, as previously described in our published work [1], and antibody information can be found in Supplementary Materials Table S6.

Data from the BALB/c mice and CD1 mice were analyzed separately. Linear (mixed) models were used. For the male BALB/c mice, all models accounted for week of assessment, radiation dose, and their two-way interaction. For CD1 mice (of both sexes), all models accounted for radiation dose, TGF-β inhibitor treatment, sex, and all two- and three-way interactions. For both CD1 and BALB/c mice, when statistically significant, we also included heart rate as a covariate on the ultrasonography measures. Normal and homogeneous variance assumptions were checked on all analyses. No substantial violations of normal assumptions were found. When there was evidence of heterogeneous variances among experimental groups, we allowed the groups’ variances to differ in the ANOVA; accommodating different variances in a linear model makes it a linear mixed model. In those instances, error degrees of freedom were estimated with the Kenward-Roger method [33]. All comparisons were made at the 0.05 significance level. When reporting summary statistics, we report the means and standard deviation (SD) estimated from the linear mixed models, since these were the quantities on which the inferences were based. The linear mixed models were fitted with the MIXED procedure in SAS/STAT^®^ software, version 9.4 (SAS Institute, Cary, NC, USA). The data and statistical code used for the analysis are available upon request.

## 3. Results

### 3.1. BALB/c Mice

Echocardiographic analysis of the male BALB/c mice at 15 weeks after GCRsim exposure are described in the Supplementary Materials, Table S1, and the results are listed in the Supplementary Materials, Tables S2 and S3. Male BALB/c mice demonstrated no change in cardiac form or function after 500 mGy GCRsim exposure. Echocardiography was not performed in the BALB/c mice evaluated at 20 weeks after GCRsim exposure.

For all the male BALB/c mice, the quantification of histological staining determined the percentage of heart tissue occupied by collagens. There was no change in collagen content at 15 or 20 weeks after GCRsim exposure (Supplementary Materials, Figure S1A). In addition, the number of capillaries per tissue area in the heart was not altered by GCRsim (Supplementary Materials, Figure S1B). Collagen type 3 is one of the most abundant collagens in the heart. Immunoblot analysis of the left ventricular tissue revealed a band of approximately 75 kDa, which we have previously observed in the mouse heart and has been identified as a truncated peptide of collagen type 3A [2,34]. The immunoblot analysis of this collagen type 3A peptide demonstrated no change after GCRsim exposure at either time point in male BALB/c mice (Supplementary Materials, Figure S1C). To detect changes in myofibroblasts after irradiation, α-smooth muscle cell actin (SMA) protein content was determined. In line with the above results, there was no change in SMA protein expression after GCRsim exposure in male BALB/c mice (Supplementary Materials, Figure S1D).

In a previous study done by our group, local X-ray exposure of the rat heart resulted in a strong correlation between collagen deposition and cardiac mast cell numbers [35]. Therefore, we assessed cardiac mast cell numbers as a possible indicator of radiation-induced tissue remodeling. There was no change in mast cell numbers or mast cell tryptase (MCT) protein expression in male BALB/c mice at 15 or 20 weeks (Supplementary Materials, Figure S2).

**Figure 1:**
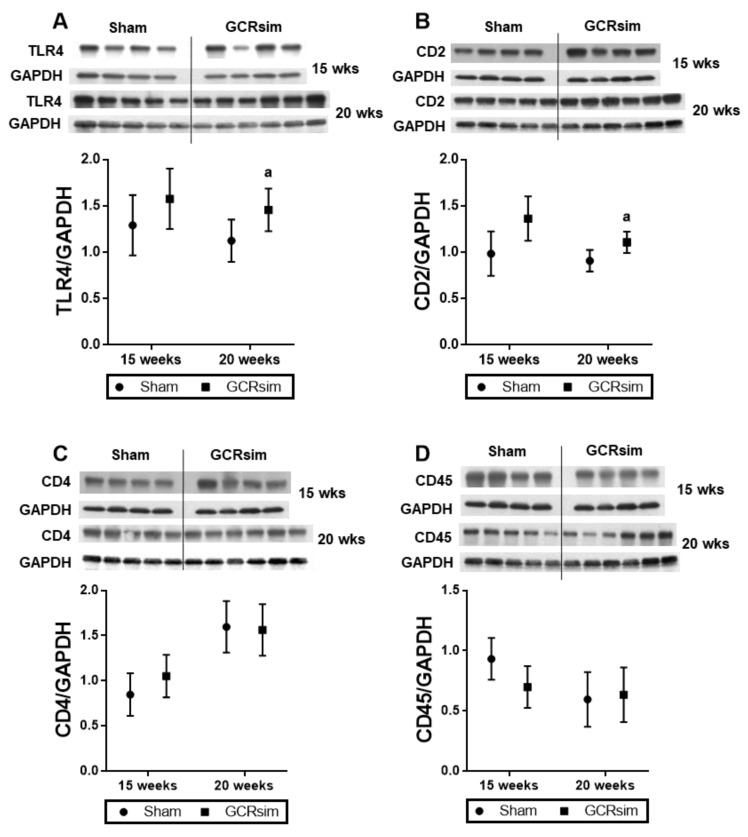
Left ventricular protein levels of immune cell markers in BALB/c mice. Western blots and analysis of proinflammatory mediator TLR4 (**A**); T cell markers CD2 (**B**), CD4 (**C**), and general leucocyte marker CD45 (**D**). Error bars indicate SD of each group estimated from the statistical model in all figures. *n* = 4–6. a indicates *p* < 0.05 compared to the time matched sham.

TLR4 is a proinflammatory mediator commonly expressed in the heart, and when in complex with MD2 it is involved in the cardiac inflammatory response to injury [36,37]. Left ventricular TLR4/MD2 protein expression was significantly increased 20 weeks after GCR exposure compared to the time matched sham (Figure 1A). Immunoblotting was used to determine if GCRsim exposure altered the left ventricular protein content of immune cell markers in male BALB/c mouse hearts. The increase in TLR4 protein expression at 20 weeks coincided only with a small but significant increase in T cell marker CD2 (Figure 1B).

**Figure 2:**
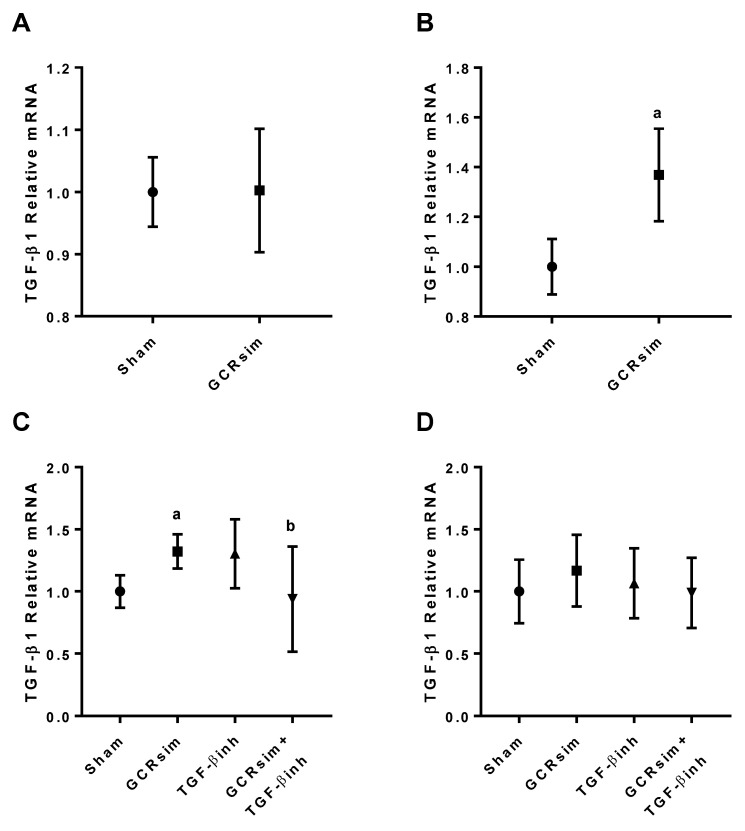
Left ventricular mRNA expression of TGF-β1 in BALB/c and CD1 mice. Relative mRNA expression of TGF-β1 in male BALB/c mice at 15 weeks (**A**) and 20 weeks (**B**). Relative mRNA expression in male CD1 mice (**C**) and female CD1 mice at 12 weeks after GCRsim exposure (**D**). a indicates *p* < 0.05 compared to sham group, b indicates *p* < 0.05 compared to GCRsim. *n*= 4–8/group.

GCRsim exposure resulted in a significant increase in the left ventricular gene expression of TGF-β1 in male BALB/c mice 20 weeks after exposure, but not at 15 weeks (Figure 2A,B).

### 3.2. CD1 Mice

There was a significant increase in TGF-β1 mRNA expression in male CD1 mice 12 weeks after GCRsim (Figure 2C) compared to the time matched sham group. In male CD1 mice, the inhibitor diet mitigated this increase in TGF-β1 expression. There was no change in TGF-β1 expression in any of the female CD1 mouse groups (Figure 2D).

Echocardiography was performed at 12 weeks after GCRsim exposure in CD1 male and female mice (described in Supplementary Materials, Table S1 and results listed in Supplementary Materials, Tables S4 and S5). Male mice exposed to 500 mGy GCRsim demonstrated no change in cardiac size or function when compared to sham mice. Administration of the TGF-β inhibitor in sham-irradiated mice resulted in significant increases in stroke volume, fractional shortening, and cardiac output compared to control. Female mice demonstrated a significantly decreased left ventricular inner diameter in diastole, end volume in diastole and left ventricular mass 12 weeks after GCRsim exposure, but the TGF-β inhibitor diet did not attenuate these changes.

**Figure 3:**
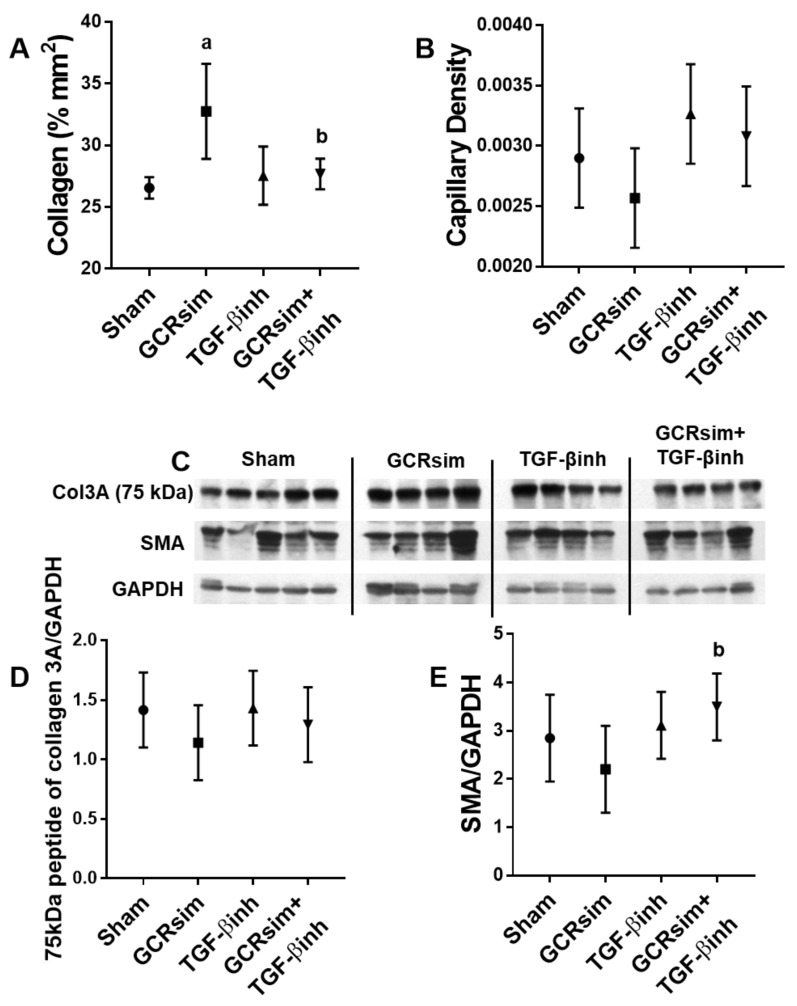
Cardiac remodeling and density of the capillary network in male CD1 mice. Histological analysis of cardiac collagen content 12 weeks after GCR exposure (**A**); number of capillaries per area of the heart (**B**); representative immunoblot of left ventricular collagen 3A (75 kDa), SMA and GAPDH (**C**); collagen 3A (75 kDa peptide) normalized to GAPDH (**D**), and SMA normalized to GAPDH (**E**). *n*= 4–5/group. a indicates *p* < 0.05 compared to the sham group, and b indicates *p* < 0.05 compared to GCRsim.

In contrast to male BALB/c mice, male CD1 mice showed a significant increase in cardiac collagen content 12 weeks after exposure to GCRsim. This increase was mitigated by the TGF-β inhibitor (Figure 3A). There was no change in the density of the cardiac capillary network (Figure 3B) or left ventricular content of the 75 kDa peptide of collagen 3A or SMA (Figure 3C–E). 

**Figure 4:**
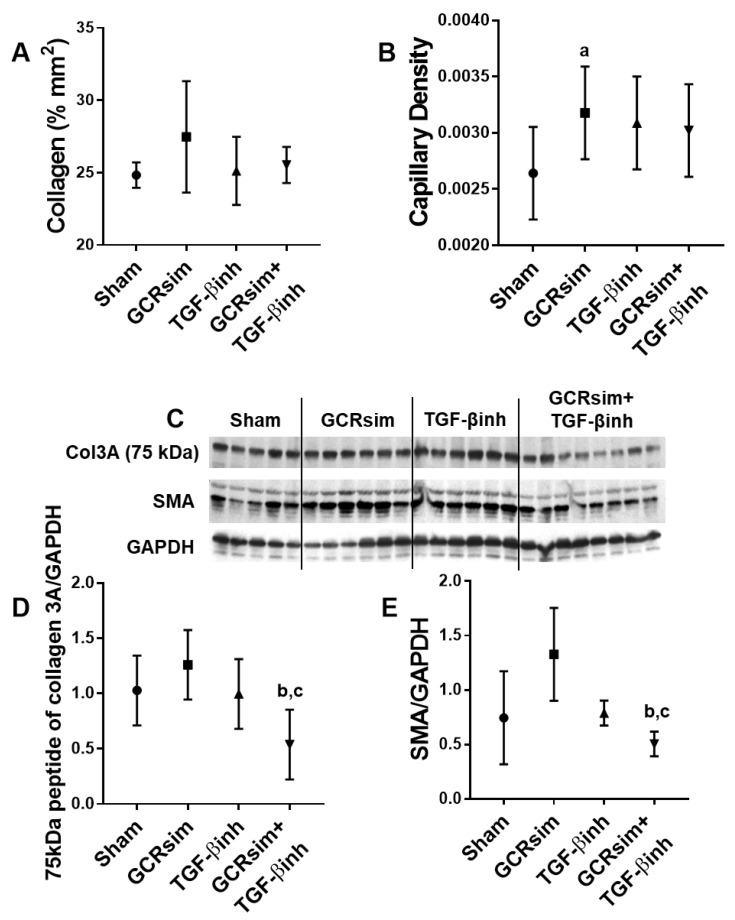
Cardiac remodeling and density of the capillary network in female CD1 mice. Histological analysis of cardiac collagen content 12 weeks after GCR exposure (**A**); number of capillaries per area of the heart (**B**); representative immunoblot of left ventricular collagen 3A (75 kDa), SMA and GAPDH (**C**); collagen 3A (75 kDa peptide) normalized to GAPDH (**D**) and SMA normalized to GAPDH (**E**). *n*= 5–9/group. a indicates *p* < 0.05 compared to the sham group, b indicates *p* < 0.05 compared to GCRsim, and c indicates *p* < 0.05 compared to the TGF-β inhibitor.

Female CD1 mice demonstrated no increase in collagen content in the heart (Figure 4A), but there was a significant increase in the density of the capillary network in the GCRsim group (Figure 4B). The combination of GCRsim exposure and the TGF-β inhibitor diet resulted in the reduced protein expression of collagen 3A peptide compared to GCRsim alone and SMA compared to GCRsim and TGF-β inhibitor alone (Figure 4C–E).

**Figure 5:**
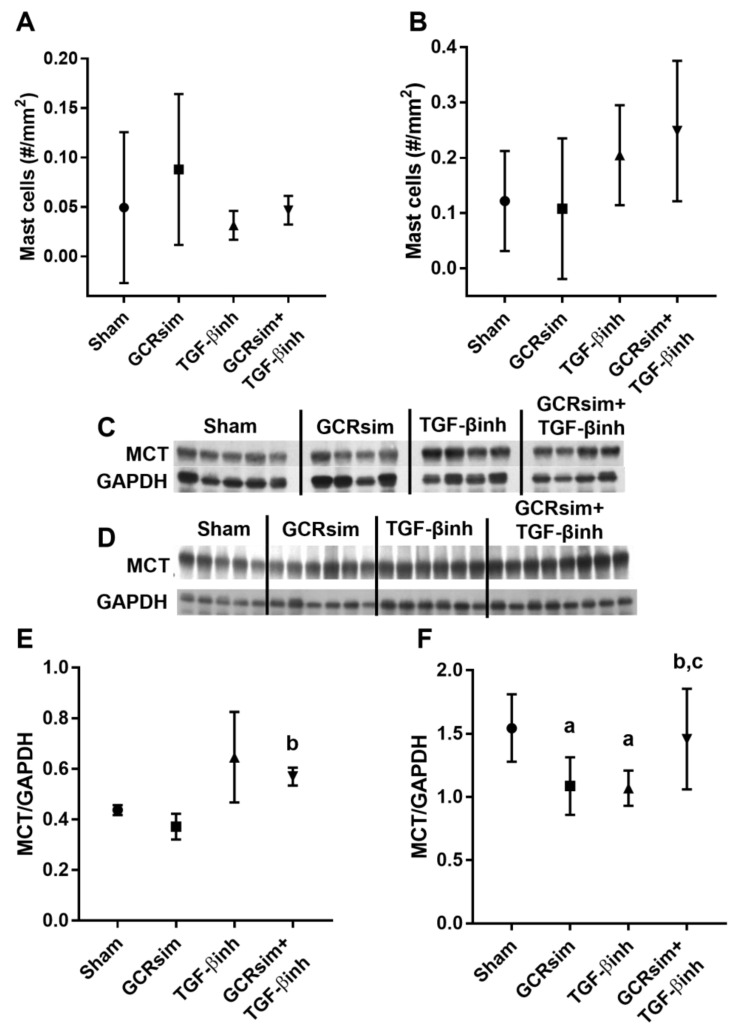
Cardiac mast cells in male and female CD1 mice. Histological analysis of cardiac mast cell numbers at 12 weeks after GCR exposure in male mice (**A**) and female mice (**B**); immunoblot and analysis of left ventricular mast cell tryptase (MCT) in male mice (**C**,**E**) and MCT content in female mice (**D**,**F**). *n* = 4–5/group in male groups and *n* = 5–9 in female groups. a indicates *p* < 0.05 compared to the sham group, b indicates *p* < 0.05 compared to GCRsim, and c indicates *p* < 0.05 compared to the TGF-β inhibitor.

There were no significant differences in mast cell numbers between groups in either male or female CD1 mice (Figure 5A,B). In male CD1 mice, GCRsim alone did not have an effect on MCT, but when GCRsim was combined with the TGF-β inhibitor, there was an increase in MCT protein expression (Figure 5C). Female CD1 mice demonstrated significantly reduced MCT protein expression after GCRsim, and the TGF-β inhibitor brought MCT protein levels back to sham levels (Figure 5D).

**Figure 6:**
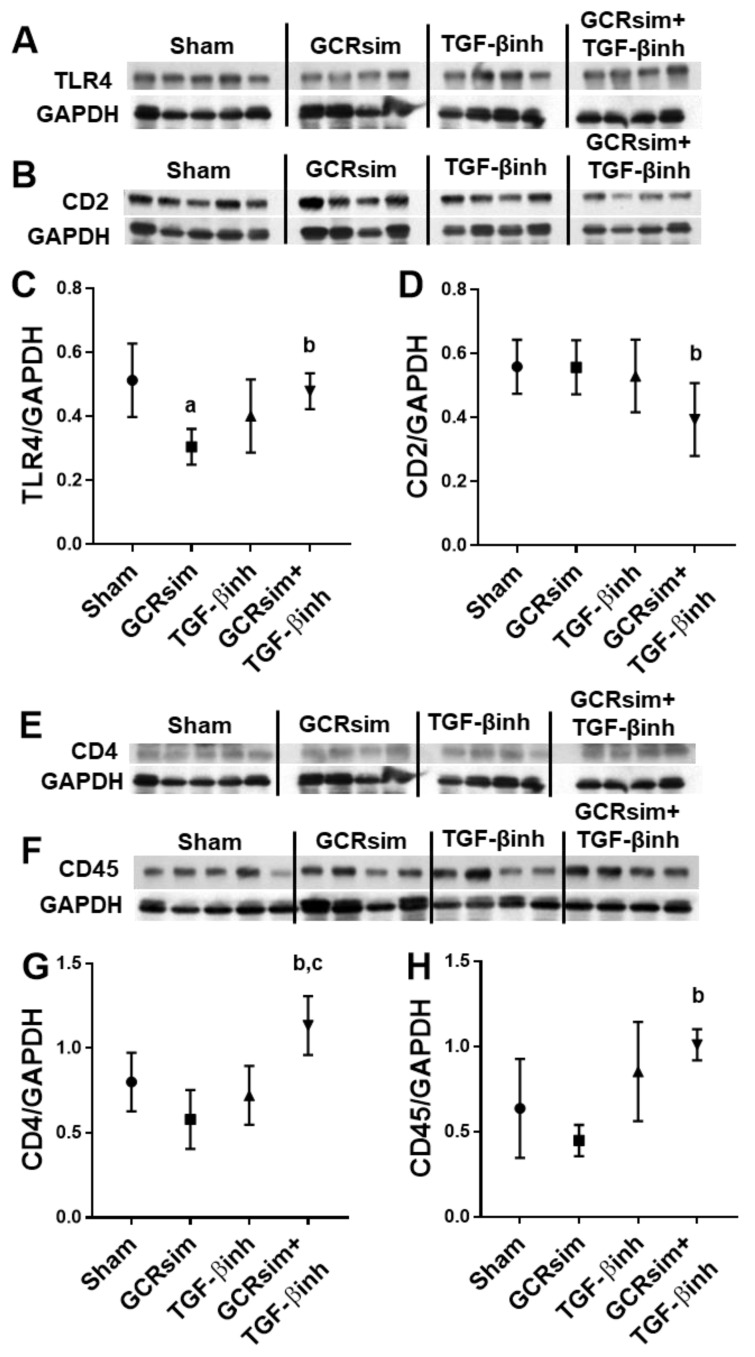
Left ventricular protein levels of immune cell markers in male CD1 mice. Western blots and analysis of proinflammatory mediator TLR4 (**A**,**C**); T cell markers CD2 (**B**,**D**) and CD4 (**E**,**G**); general leucocyte marker CD45 (**F**,**H**). *n* = 4–5/group. a indicates *p* < 0.05 compared to the sham group, b indicates *p* < 0.05 compared to GCRsim, and c indicates *p* < 0.05 compared to TGF-β inhibition.

**Figure 7:**
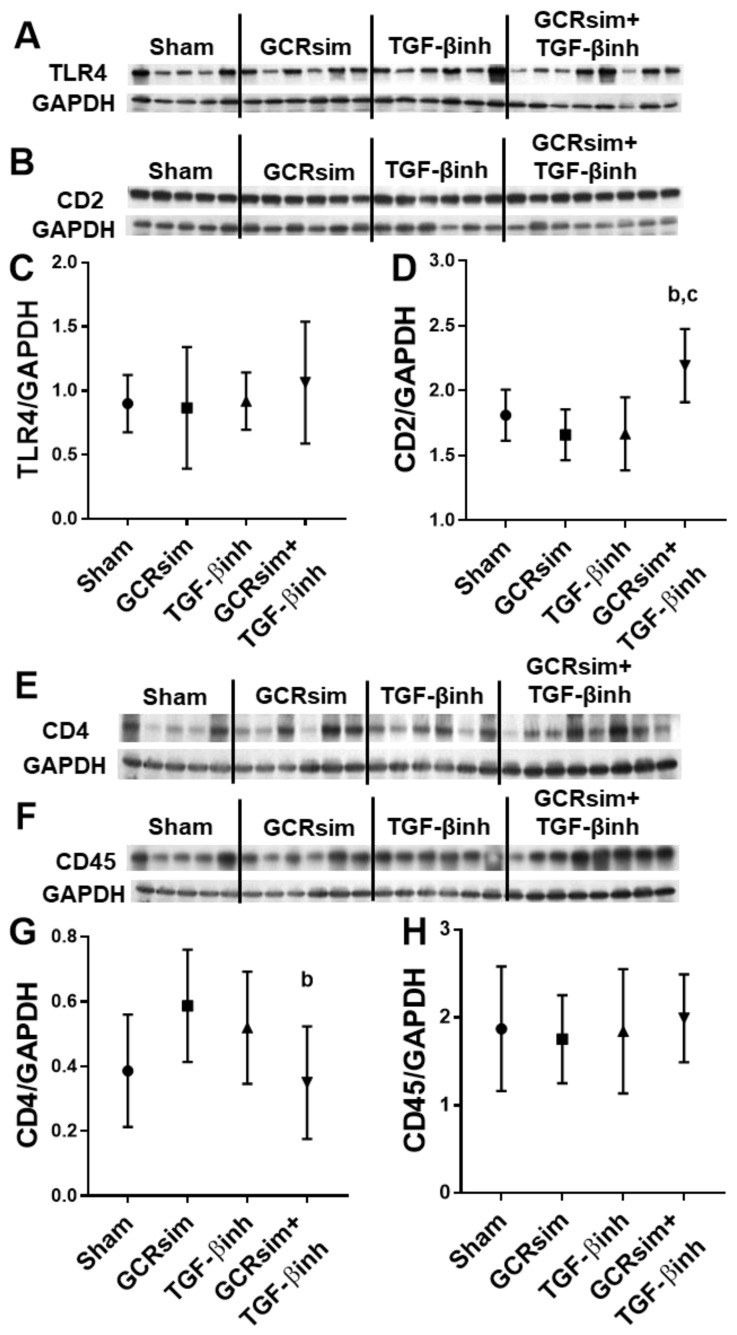
Left ventricular protein levels of immune cell markers in female CD1 mice. Western blots and analysis of proinflammatory mediator TLR4 (**A**,**C**); T cell markers CD2 (**B**,**D**) and CD4 (**E**,**G**); general leucocyte marker CD45 (**F**,**H**). *n* = 5–8. b indicates *p* < 0.05 compared to GCRsim, and c indicates *p* < 0.05 compared to the TGF-β inhibition.

In contrast to male BALB/c mice, male CD1 mice showed a significant decrease in left ventricular TLR4 expression 12 weeks after GCRsim (Figure 6A,C). Nonetheless, GCRsim had no effect on T cell markers CD2 and CD4 or leucocyte marker CD45 (Figure 6B,D–H). Interestingly, the combination of GCRsim and TGF-β inhibitor had a significant effect on the expression of each of the markers when compared to GCRsim alone. In female CD1 mice, GCRsim exposure did not alter TLR4 expression or cardiac immune cell markers. However, GCRsim + TGF-β inhibition resulted in significantly decreased CD4 protein content compared to the GCRsim group (Figure 7).

## 4. Discussion

This study aimed to provide insights into the effects of GCRsim exposure on the heart. First, we examined the effects of simplified (5-ion) GCRsim on adult male BALB/c mice. In a second study, we utilized adult male and female mice of an outbred strain, CD1, to again determine the cardiac effects of simplified GCRsim exposure, but also to test whether inhibiting TGF-β signaling would be a potential countermeasure against these effects. 

Ultrasonography revealed no change in cardiac form or function in male mice of either strain after GCRsim exposure. The use of ultrasound did identify significant decreases in left ventricular mass and left ventricular inner diameter in diastole and end diastolic volume after GCRsim exposure in female CD1 mice. In addition, there was a significant increase in the density of the capillary network in the myocardium of these irradiated female CD1 mice, which may be explained at least in part by the decrease in their left ventricular mass. Our previous studies using single ion beams have shown changes in cardiac ejection fraction and fractional shortening in male C57BL/6J mice [1,2] but not in female C57BL/6J mice [6], indicating that sex differences in radiation response in the heart can occur. Indeed, there is an increasing number of studies demonstrating sex differences after GCR exposure [6,38,39]. However the exact mechanisms of how GCRsim effects the cardiovascular system of males and females differently remains unclear. Estrogen and estrogen receptors have been shown to be cardioprotective, which could be due to their antioxidant and anti-inflammatory effects [40,41]. Future studies should investigate the mechanisms behind sex differences seen after GCRsim exposure.

In our previous studies in C57BL/6 mice, changes in cardiac function parameters were not seen until 9 months after irradiation [1]. Moreover, a recent study of the effects of the 5-ion GCRsim exposure (1.5 Gy) in adult male C57BL/6 mice also demonstrated minor changes in cardiac ultrasound parameters 12 months after irradiation [42]. In the present study, ultrasonography was performed 12–20 weeks after GCRsim exposure. We cannot exclude that a longer follow-up of the mice would have resulted in more significant changes in cardiac function. 

TGF-β inhibition has previously been shown to significantly reduce fibrosis in mice exposed to high dose low-LET radiation [31]. Therefore, to assess tissue remodeling, we determined collagen content and the expression of myofibroblasts marker SMA in the heart. Male BALB/c mice demonstrated no change in collagen content or SMA due to GCRsim. However, male CD1 mice exposed to GCRsim showed a significant increase in collagen percentage in the heart, although this was not seen in female CD1 mice. While Yan et al. demonstrated cardiac fibrosis after proton and ^56^Fe (150 mGy, 1 GeV/n) exposure in male C57BL/6NT mice [7], our previous studies using ^16^O (100 mGy-1 Gy, 600 MeV/n) in male and female C57BL/6J mice did not result in increased cardiac collagen deposition [1,6]. Overall, these studies demonstrate the importance of studying the effects of simulated GCR in multiple animal models and animal strains, since they may have different outcomes. In the present study, although female CD1 mice did not show an increase in cardiac collagens after irradiation, there was a significant decrease in a 75 kDa peptide of collagen type 3A in the GCRsim + TGF-β inhibitor group compared to the GCRsim or TGF-β inhibitor alone. These results suggest that TGF-β is involved in maintaining normal cardiac collagen levels in the GCRsim exposed heart. 

In addition to collagen content, we also determined mast cell numbers as another indication of cardiac remodeling. Mast cells can regulate cardiac tissue remodeling by producing pro- and anti-fibrogenic mediators [43]. The heart normally contains a small number of mast cells [44], and our previous study demonstrated that increased mast cells were correlated with increased fibrosis [35]. In the present study there was no change in mast cell numbers in either mouse strain despite increased collagen content in male CD1 mice. However, there was a significant decrease in female CD1 MCT protein. The inhibition of TGF-β resulted in an increase in MCT protein in GCRsim mice of both sexes. TGF-β is known to regulate MCT expression by mast cells [45,46], but the role of this TGF-β—MCT axis in cardiovascular function remains to be studied.

In addition to mast cell numbers, we examined whether GCR exposure influenced the immune cells in the heart by measuring the left ventricular protein content of CD2, CD4, CD45, and inflammatory marker TLR4. Male BALB/c mice demonstrated a small but significant increase in left ventricular CD2 and TLR4 15 weeks after GCRsim, whereas male CD1 mice demonstrated a small but significant decrease in TLR4, again pointing to the importance of studying multiple animal strains. While there are few articles investigating the health effects of mixed ion GCRsim, single ion radiation exposure has been studied more frequently and shown to influence immune cells and inflammatory mediators [1,2,39]. Our previous study utilizing ^16^O (250 mGy, 600 MeV/n) demonstrated an increase in left ventricular expression of the CD2 and CD68 protein markers 2 weeks and 3 months after exposure in C57Bl/6 male mice [1]. Other studies in animal models of single ion exposure have also shown increased immune cell markers [7,9] and inflammatory cytokines [4] in the heart. How HZE ion exposure influences cardiac immune cell infiltration in male and female mice is not fully understood. 

The GCRsim-induced decrease in TLR4 was mitigated by the TGF-β inhibitor diet. In addition, the combination of GCRsim and the TGF-β inhibitor resulted in significant changes in left ventricular CD2, CD4 and CD45 compared to GCRsim alone. This interaction of GCRsim and TGF-β inhibition was also seen on CD2 and CD4 in the female mice, suggesting that the TGF-β pathway may play a role in regulating immune cells in the heart after GCRsim exposure. At low doses, low-LET radiation has been known to alter immune responses via TGF-β [47,48]. Interestingly, one group investigating ^4^He (50 or 300 mGy, 400 MeV/n) exposure in the mouse brain found that female C57BL/6 mice had significant resistance to adverse neurocognitive effects compared to male mice of the same strain, which may have been due to differences in inflammatory responses between the two sexes [39]. Further investigation into potential differences between male and female immune cells in the heart in response to GCRsim is required. 

Although 500 mGy GCRsim is a total dose that may be encountered during a deep space mission, this dose was given in about 4 h, whereas the dose rate of HZE particles in space is much lower [49,50]. Moreover, while the GCRsim reflects multiple ions that are found in the deep space radiation environment, NSRL also provides a full spectrum 30-ion beam GCR simulator. Further studies are required to confirm the results in this study, using lower dose rates and other GCR simulations. During space missions, astronauts will not only be subject to GCR exposure, but will be in microgravity, which also contributes to cardiovascular health concerns. Studies with rodents that are exposed to GCRsim while simultaneously submitted to hindlimb unloading to model microgravity are underway. Such studies are important in providing additional insight in the health risks of space travel and to develop and test countermeasures that will allow astronauts to explore deep space safely. 

## 5. Conclusions

In conclusion, the exposure of adult male BALB/c mice and adult male and female CD1 mice to 500 mGy GCRsim did not cause major changes in cardiac form or function. Male CD1 mice demonstrated some evidence of cardiac remodeling which the TGF-β inhibitor diet attenuated. In both male and female CD1 mice, the combination of GCRsim and TGF-β inhibition had effects on collagens and immune cell markers that were not observed after GCRsim or TGF-β inhibition alone. This interaction suggests that TGF-β may play a role in maintaining collagens and immune cells in the irradiated heart. In future studies, the post-irradiation follow-up time should be extended, since we know that cardiovascular radiation effects occur late after exposure. In addition to more accurately representing the radiation seen outside LEO, the effects of chronic exposure to low dose rate GCR should be investigated when the technology for such simulations becomes available. Lastly, more in-depth comparisons between male and female animals will aid in understanding differences between the sexes.

## Data Availability

Data available upon request.

## Supplementary Materials

The following supporting information can be downloaded at: https://www.mdpi.com/article/10.3390/life13030795/s1, Table S1: Description of parameters obtained with ultrasonography; Table S2: Results of cardiac ultrasound after sham (0 mGy) and GCRsim irradiation (500 mGy) in male BALB/c mice; Table S3: Results of pulsed wave Doppler of the mitral valve after sham (0 mGy) and GCRsim irradiation (500 mGy) in male BALB/c mice.; Table S4: Results of cardiac ultrasound after sham-irradiation + vehicle (0 mGy), sham-irradiated + TGFβ inhibitor (0 mGy + TGFβinh), GCRsim irradiation (500 mGy), or GCRsim irradiation + TGFβ inhibitor (GCRsim + TGFβinh) in male and female CD1 mice; Table S5: Results of pulsed wave Doppler of the mitral valve after sham-irradiation + vehicle (0 mGy), sham-irradiated + TGFβ inhibitor (0 mGy + TGFβinh), GCRsim irradiation (500 mGy), or GCRsim irradiation + TGFβ inhibitor (GCRsim + TGFβinh) in CD1 male and female mice; Table S6: Antibodies used in western blot technique. Figure S1: Cardiac remodeling and density of the capillary network in male BALB/c mice; Figure S2: Cardiac mast cells in male BALB/c mice.
