# Supplementary material for: Effects of Simulated 5-Ion Galactic Cosmic Radiation on Function and Structure of the Mouse Heart
Source: Life (Basel). 2023 Mar 15;13(3):795. doi: 10.3390/life13030795 (PMC10057791; doi:10.3390/life13030795)
Supplement: Supplementary file 1 [file life-13-00795-s001.zip › life-2250050-supplementary.pdf]

Supplemental Material

Effects of simulated 5-ion galactic cosmic radiation on function and structure of the mouse heart

Table S1. Description of parameters obtained with ultrasonography

Parameter	Description
Cardiac ultrasound (parasternal short axis M-mode)	
LVAW_d	Left ventricle anterior wall thickness in diastole (mm)
LVAW_s	Left ventricle anterior wall thickness in systole (mm)
LVID_d	Left ventricle internal diameter in diastole (mm)
LVID_s	Left ventricle internal diameter in systole (mm)
LVPW_d	Left ventricle posterior wall thickness in diastole (mm)
LVPW_s	Left ventricle posterior wall thickness in systole (mm)
LVmass	Left ventricle mass (mg)
D_s	Diameter of left ventricle at systole (mm)
D_d	Diameter of left ventricle at diastole (mm)
V_s	End systolic volume (mm^3)
V_d	End diastolic volume (mm^3)
SV	Stroke volume, (difference between V_d and V_s) (µl)
EF	Ejection fraction (V_d – V_s / V_d)*100) (%)
FS	Fractional shortening (D_d – D_S) / D_d)*100 (%)
CO	Cardiac output (SV*heart rate) (ml/min)
Abdominal aorta pulsed-wave Doppler	
Mean Vel	Mean velocity (mm/s), obtained from VTI tracing
Peak Vel	Peak velocity (mm/s), obtained from VTI tracing
VTI	Velocity time integral (mm), obtained from VTI tracing
Four-chamber view pulsed-wave Doppler	
MVE	Mitral valve E wave velocity (mm/s)
MVA	Mitral valve A wave velocity (mm/s)
MVEA	Mitral valve E/A

Table S2. Results of cardiac ultrasound after sham (0 cGy) and GCRsim irradiation (50 cGy) in male BALB/c mice.

Parameter	Treatment	Average ± SD
LVAW_d (mm)	0 cGy	1.0 ± 0.1
	GCRsim	0.9 ± 0.1
LVAW_s (mm)	0 cGy	1.4 ± 0.2
	GCRsim	1.3 ± 0.2
LVID_d (mm)	0 cGy	4.4 ± 0.2
	GCRsim	4.3 ± 0.2
LVID_s (mm)	0 cGy	3.3 ± 0.2
	GCRsim	3.2 ± 0.2
LVPW_d (mm)	0 cGy	0.9 ± 0.9
	GCRsim	0.9 ± 0.9
LVPW_s (mm)	0 cGy	1.1 ± 0.1
	GCRsim	1.1 ± 0.1
D_s (mm)	0 cGy	3.2 ± 0.2
	GCRsim	3.0 ± 0.2
D_d (mm)	0 cGy	4.5 ± 0.2
	GCRsim	4.2 ± 0.2
V_s (mm^3)	0 cGy	40.0 ± 7.7
	GCRsim	36.9 ± 7.7
V_d (mm^3)	0 cGy	90.5 ± 9.7
	GCRsim	80.8 ± 9.7
SV (µl)	0 cGy	49.4 ± 7.0
	GCRsim	45.2 ± 7.0
EF (%)	0 cGy	55.3 ± 3.7
	GCRsim	55.6 ± 8.7
FS (%)	0 cGy	28.7 ± 2.4
	GCRsim	29.1 ± 5.7
CO (ml/min)	0 cGy	19.9 ± 3.4
	GCRsim	19.0 ± 3.4

Values indicate average ± SD, n=9 mice in 0 cGy and n=8 mice in GCRsim. $p < 0.05$ compared to time-matched 0 cGy.

Table S3. Results of pulsed wave Doppler of the mitral valve after sham (0 cGy) and GCRsim irradiation (50 cGy) in male BALB/c mice.

Parameter	Treatment	Average ± SD
MVE (mm/s)	0 cGy	402.4 ± 66.4
	GCRsim	405.1 ± 66.4
MVA (mm/s)	0 cGy	767.2 ± 115.7
	GCRsim	715.7 ± 115.7
MVEA (mm/s)	0 cGy	2.1 ± 0.5
	GCRsim	1.7 ± 0.5

Values indicate average ± SD, n=9 mice in 0 cGy and n=8 mice in GCR. $p < 0.05$ compared to time-matched 0 cGy.

Table S4. Results of cardiac ultrasound after sham-irradiation + vehicle (0 cGy), sham-irradiated + TGFβ inhibitor (0 cGy+TGFβinh), GCRsim irradiation (50 cGy), or GCRsim irradiation + TGFβ inhibitor (GCRsim+TGFβinh) in male and female CD1 mice.

Parameter	Treatment	Females Average ± SD	Males Average ± SD
LVAW_d (mm)	0 cGy	1.1 ± 0.2	1.1 ± 0.1
	0 cGy+TGFβinh	0.9 ± 0.2	1.1 ± 0.1
	GCRsim	1.0 ± 0.1	1.1 ± 0.2
	GCRsim+TGFβinh	0.9 ± 0.1	1.1 ± 0.2
LVAW_s (mm)	0 cGy	1.6 ± 0.2	1.5 ± 0.1
	0 cGy+TGFβinh	1.4 ± 0.2	1.6 ± 0.1
	GCRsim	1.5 ± 0.1	1.6 ± 0.3
	GCRsim+TGFβinh	1.4 ± 0.1[c]	1.6 ± 0.3
LVID_d (mm)	0 cGy	4.2 ± 0.3	4.6 ± 0.2
	0 cGy+TGFβinh	4.2 ± 0.3	4.7 ± 0.2
	GCRsim	3.9 ± 0.2[a]	4.7 ± 0.5
	GCRsim+TGFβinh	4.1 ± 0.2	4.6 ± 0.5
LVID_s (mm)	0 cGy	2.9 ± 0.4	3.5 ± 0.3
	0 cGy+TGFβinh	2.8 ± 0.4	3.3 ± 0.3
	GCRsim	2.7 ± 0.2	3.5 ± 0.5
	GCRsim+TGFβinh	2.9 ± 0.2[c]	3.2 ± 0.5
LVPW_d (mm)	0 cGy	0.9 ± 0.1	1.0 ± 0.1
	0 cGy+TGFβinh	0.9 ± 0.1	0.9 ± 0.1
	GCRsim	0.9 ± 0.1	1.0 ± 0.1
	GCRsim+TGFβinh	0.9 ± 0.1	1.1 ± 0.1[b]
LVPW_s (mm)	0 cGy	1.2 ± 0.2	1.2 ± 0.2
	0 cGy+TGFβinh	1.3 ± 0.2	1.3 ± 0.2
	GCRsim	1.3 ± 0.2	1.3 ± 0.2
	GCRsim+TGFβinh	1.2 ± 0.2	1.4 ± 0.2
LVmass (mg)	0 cGy	138.8 ± 24.3	163.8 ± 16.8
	0 cGy+TGFβinh	126.7 ± 24.3	165.6 ± 16.8
	GCRsim	114.7 ± 12.2[a]	172.7 ± 36.5
	GCRsim+TGFβinh	118.2 ± 12.2	182.7.7 ± 36.5
D_s (mm)	0 cGy	2.9 ± 0.5	3.3 ± 0.3
	0 cGy+TGFβinh	2.8 ± 0.5	3.1 ± 0.3
	GCRsim	2.6 ± 0.2	3.4 ± 0.6
	GCRsim+TGFβinh	2.8 ± 0.2[c]	3.1 ± 0.6

D_d (mm)	0 cGy	4.2 ± 0.3	4.6 ± 0.2
	0 cGy+TGFβinh	4.2 ± 0.3	4.7 ± 0.2
	GCRsim	4.0 ± 0.2	4.8 ± 0.5
	GCRsim+TGFβinh	4.1 ± 0.2	4.6 ± 0.5
V_s (mm^3)	0 cGy	33 ± 12.2	49.7 ± 9.6
	0 cGy+TGFβinh	31.6 ± 12.2	43.2 ± 9.6
	GCRsim	26.2 ± 6.0	45.9 ± 14.6
	GCRsim+TGFβinh	32.9 ± 6.0[c]	43.2 ± 14.6
V_d (mm^3)	0 cGy	79.8 ± 13.9	98.1 ± 7.9
	0 cGy+TGFβinh	80.5 ± 13.9	103.7 ± 7.9
	GCRsim	67.4 ± 10.0[a]	96.5 ± 19.5
	GCRsim+TGFβinh	75.9 ± 10.0	97.5 ± 19.5
SV (μl)	0 cGy	46.4 ± 9.4	55.4 ± 9.4
	0 cGy+TGFβinh	51.0 ± 9.4	65.1 ± 9.4[a]
	GCRsim	46.3 ± 9.4	55.2 ± 9.4
	GCRsim+TGFβinh	46.3 ± 9.4	57.9 ± 9.4
EF (%)	0 cGy	57.5 ± 10.5	49.9 ± 7.3
	0 cGy+TGFβinh	62.0 ± 10.5	58.2 ± 7.3
	GCRsim	61.9 ± 7.5	52.5 ± 9.3
	GCRsim+TGFβinh	54.7 ± 7.5[c]	56.2 ± 9.3
FS (%)	0 cGy	30.5 ± 8.3	25.3 ± 8.3
	0 cGy+TGFβinh	35.9 ± 8.3	31.0 ± 8.3[a]
	GCRsim	33.0 ± 8.3	27.1 ± 8.3
	GCRsim+TGFβinh	29.6 ± 8.3	29.6 ± 8.3
CO (ml/min)	0 cGy	22.3 ± 4.8	24.5 ± 4.8
	0 cGy+TGFβinh	22.5 ± 4.8	29.7 ± 4.8[a]
	GCRsim	20.1 ± 4.8	26.3 ± 4.8
	GCRsim+TGFβinh	20.7 ± 4.8	26.3 ± 4.8

Values indicate average ± SD, *n*=8 mice in 0 cGy, *n*=9 mice in 0 cGy+TGFβinh, *n*=8 mice in GCRsim, and *n*=9 mice in the GCRsim+TGFβinh groups. $p < 0.05$. a indicates significant difference when compared to 0 cGy, b indicates significant difference when compared to 0 cGy+TGFβinh, and c indicates significant difference when compared to GCRsim.

Table S5. Results of pulsed wave Doppler of the mitral valve after sham-irradiation + vehicle (0 cGy), sham-irradiated + TGFβ inhibitor (0 cGy+TGFβinh), GCRsim irradiation (50 cGy), or GCRsim irradiation + TGFβ inhibitor (GCRsim+TGFβinh) in CD1 male and female mice.

Parameter		Females Average ± SD	Males Average ± SD
MVE (mm/s)	0 cGy	641.3 ± 112.6	687.2 ± 112.6
	0 cGy+TGFβinh	559.9 ± 112.6	683.6 ± 112.6
	GCRsim	542.1 ± 112.6	628.7 ± 112.6
	GCRsim+TGFβinh	648.2 ± 112.6[c]	597.4 ± 112.6
MVA (mm/s)	0 cGy	350.3 ± 93.5	401.3 ± 93.5
	0 cGy+TGFβinh	272.2 ± 87.7	373.6 ± 87.7
	GCRsim	293.6 ± 93.5	365.3 ± 93.5
	GCRsim+TGFβinh	385.0 ± 87.7[b,c]	318.8 ± 87.7
MVEA (mm/s)	0 cGy	2.1 ± 0.4	1.7 ± 0.4
	0 cGy+TGFβinh	2.1 ± 0.4	2.0 ± 0.4
	GCRsim	1.8 ± 0.4	2.0 ± 0.4
	GCRsim+TGFβinh	1.8 ± 0.4	1.9 ± 0.4

Values indicate average ± SD, n=8-9 mice in 0 cGy, n=9-10 mice in 0 cGy+TGFβinh, n=8-10 mice in GCRsim, and n=9-12 mice in the GCRsim+TGFβinh groups. $p < 0.05$. a indicates significant difference when compared to 0 cGy, b indicates significant difference when compared to 0 cGy+ TGFβinh, and c indicates significant difference when compared to GCRsim.

Table S6. Antibodies used in western blot technique.

Antibody	Company and Dilution
Rat anti- α-SMA	Abcam, 1:4,000
Rabbit anti- TLR4-MD2	Invitrogen, 1:5,000
Rabbit anti-collagen type III	Santa Cruz, 1:1,000
Rabbit anti-mast cell tryptase	Santa Cruz, 1:20,000
Rabbit anti-CD2	Santa Cruz, 1:3,000
Mouse anti-CD4	Santa Cruz, 1:1,000
Rat anti-C45	Santa Cruz, 1:40,000
Mouse anti-GAPDH	Santa Cruz, 1:20,000
Goat Anti-Rabbit IgG	Cell Signaling Technologies, 1:10,000
Goat Anti-Rat IgG	Santa Cruz, 1:30,000
Goat Anti-Mouse IgG	Jackson Immunoresearch, 1:10,000 or 1:20,000

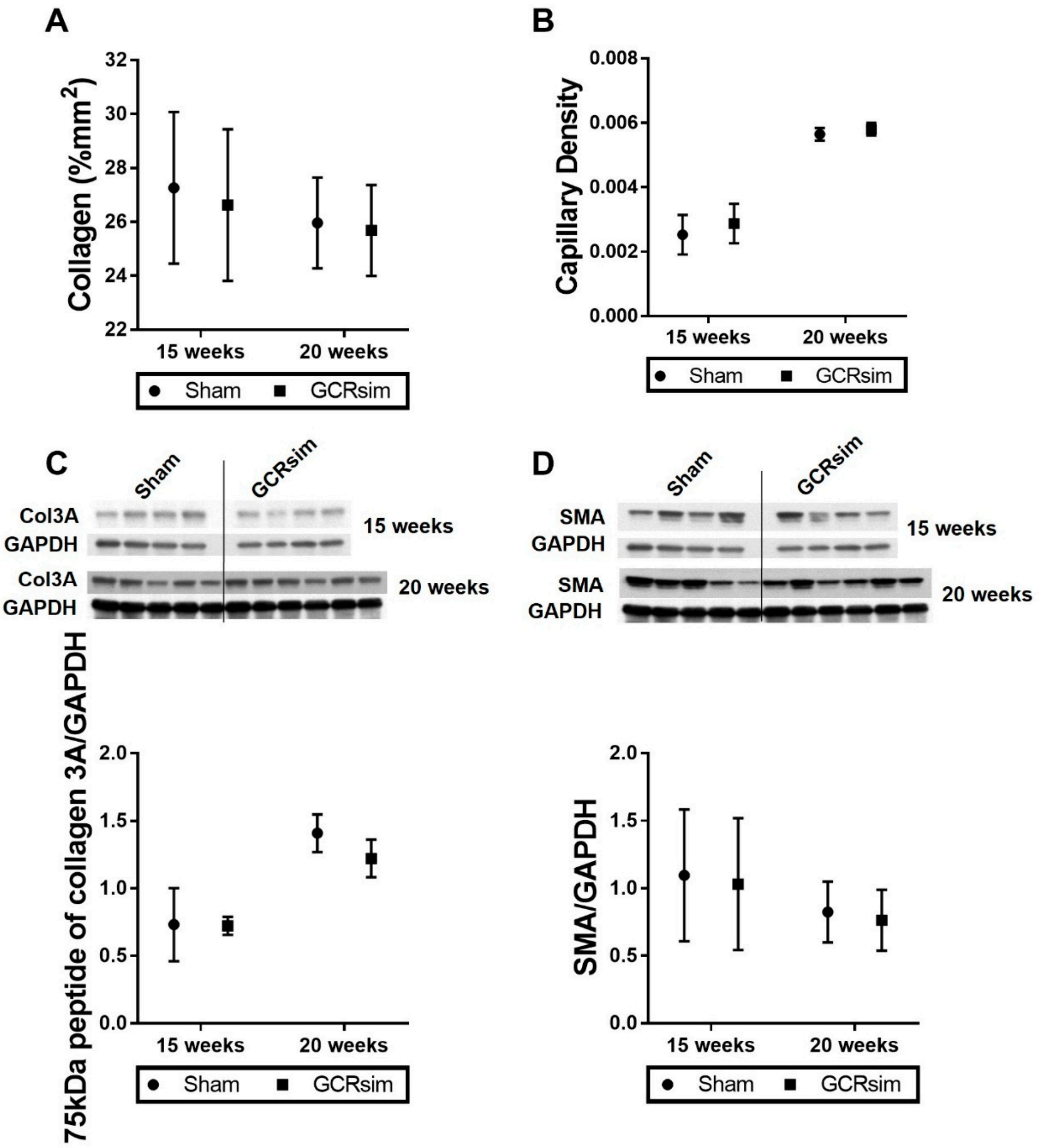

Fig S1: Cardiac remodeling and density of the capillary network in male BALB/c mice. Histological analysis of cardiac collagen content 15 weeks and 20 weeks after GCRsim exposure (A); number of capillaries per area of the heart (B); western blot and analysis of collagen 3A (75 kDa peptide) (C) and α-smooth muscle cell actin (SMA) (D). Error bars indicate SD of each group estimated from the statistical model. There were no significant differences between groups. *n*= 4-6/group.

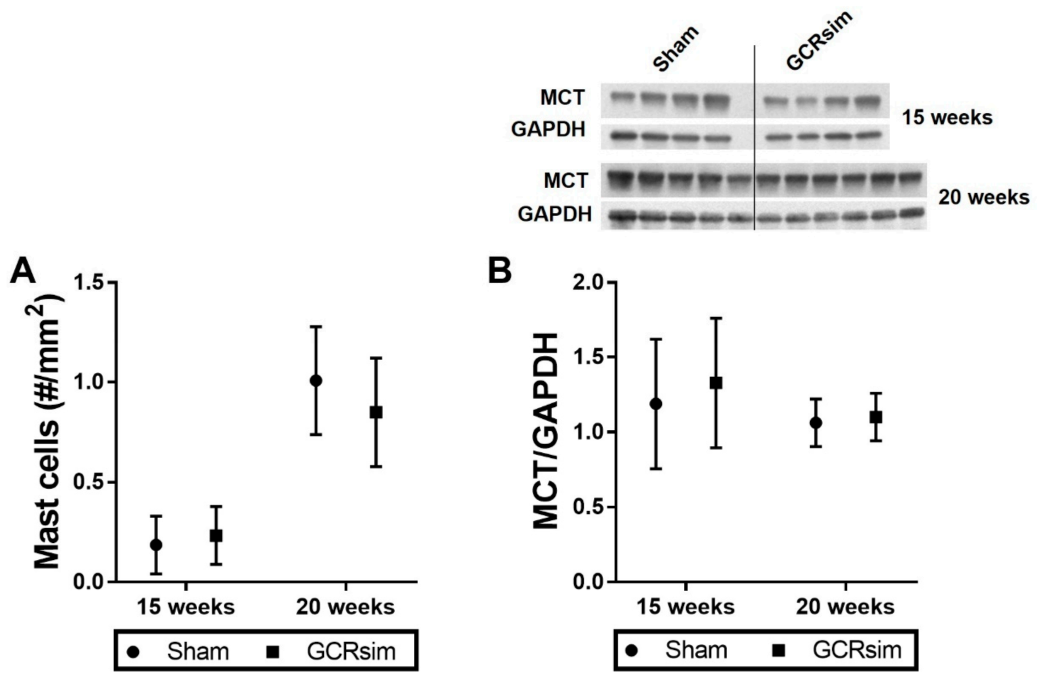

Fig S2: Cardiac mast cells in male BALB/c mice. Histological analysis of cardiac mast cell numbers at 15 weeks and 20 weeks after GCRsim exposure (A); western blot and analysis of left ventricular mast cell tryptase (MCT) (B). Error bars indicate SD of each group estimated from the statistical model. There were no significant differences between groups. *n*= 4-6/group.
